# Development of Synthetic DNA Circuit and Networks for Molecular Information Processing

**DOI:** 10.3390/nano11112955

**Published:** 2021-11-04

**Authors:** Yongpeng Zhang, Yuhua Feng, Yuan Liang, Jing Yang, Cheng Zhang

**Affiliations:** 1 School of Control and Computer Engineering, North China Electric Power University, Beijing 102206, China; alzghy@163.com (Y.Z.); 15513092465@163.com (Y.F.); 18811658276@163.com (Y.L.); 2 Department of Computer Science and Technology, School of Electronics Engineering and Computer Science, Peking University, Beijing 100871, China

**Keywords:** synthetic DNA circuit, DNA strand displacement, DNA self-assembly, DNA networks, DNA computing

## Abstract

Deoxyribonucleic acid (DNA), a genetic material, encodes all living information and living characteristics, e.g., in cell, DNA signaling circuits control the transcription activities of specific genes. In recent years, various DNA circuits have been developed to implement a wide range of signaling and for regulating gene network functions. In particular, a synthetic DNA circuit, with a programmable design and easy construction, has become a crucial method through which to simulate and regulate DNA signaling networks. Importantly, the construction of a hierarchical DNA circuit provides a useful tool for regulating gene networks and for processing molecular information. Moreover, via their robust and modular properties, DNA circuits can amplify weak signals and establish programmable cascade systems, which are particularly suitable for the applications of biosensing and detecting. Furthermore, a biological enzyme can also be used to provide diverse circuit regulation elements. Currently, studies regarding the mechanisms and applications of synthetic DNA circuit are important for the establishment of more advanced artificial gene regulation systems and intelligent molecular sensing tools. We therefore summarize recent relevant research progress, contributing to the development of nanotechnology-based synthetic DNA circuits. By summarizing the current highlights and the development of synthetic DNA circuits, this paper provides additional insights for future DNA circuit development and provides a foundation for the construction of more advanced DNA circuits.

## 1. Introduction

DNA, carrying the genetic information necessary for the synthesis of RNA and proteins, is an essential biological molecule for controlling various complicated life functions. In cell, transcription activities of genes are controlled by DNA signaling circuits, where specific DNA signals are manipulated, causing them to interact with each other, thus regulating gene networks. Recently, various artificial DNA circuits have been established and widely applied to many fields such as medical diagnosis [1,2,3], molecular detection, and information processing [4,5,6,7,8,9,10]. Particularly, synthetic DNA circuits, designed and constructed in vitro, perform an important role in effectively controlling the gene networks in cell [11,12,13]. Synthetic DNA circuits have been demonstrated as possessing superiority in simulating and regulating DNA signaling, due to the properties of programmability and easy operation [14,15,16,17,18]. More importantly, synthetic DNA circuits have the potential to promote complex biological information processes and provide a new way to achieve gene analysis and molecular information processing [19,20,21].

Using predesigned specific base pair recognition, synthetic DNA circuits can modulate complex gene networks to implement diverse biofunctions. Recently, a variety of bioengineering and biocomputing functions have been regulated by varying the architectures and integrations of DNA circuits, such as their signal simulation [5,22], the molecular switch, catalytic cycle, cascade amplification [23,24,25,26,27,28,29,30], and logic gates [31,32,33,34,35,36,37]. In fact, most of the DNA circuits are implemented and regulated for the DNA strand displacement, whereby the longer DNA strand is able to hybridize with the complementary strand to displace the shorter one [38,39,40]. Through a DNA strand displacement reaction (SDR), a synthetic DNA circuit can be used to precisely regulate complex gene networks and molecular biosystems, e.g., DNA neural network systems that are constructed to implement pattern recognition [41]. In addition, by taking advantage of both DNA SDR and enzyme assisted reactions, more complex logic functions can be realized, such as the multilayer DNA circuit-based logic gate that has been established by Song, T et. al. to calculate a four-digit input square root in binary [42]. Moreover, by using a synthetic DNA circuit, the dynamic nanoparticle self-assembly process can be adequately controlled to construct specific nanostructures [43,44,45,46]. For example, the spatial arrangement of gold nanoparticles can be controlled by DNA circuits [47,48]. In recent years, synthetic DNA circuits have been developed rapidly and have attracted more attention. It has been well demonstrated by recent studies that synthetic DNA circuits are useful and versatile tools, that have further potential applications in the fields of biosensing, diagnostics, biocomputing, nanorobots and biosystems.

In this review article, we summarized the recent developments and applications of DNA circuits. We will focus on the design principles of synthetic DNA circuits, combined with enzymes, nanoparticles and DNA origami etc. Based on the construction methods and materials of the synthetic DNA circuits, we divide the DNA circuits into five categories: (1) Cascading DNA circuits, with signals transmitted via upstream units to the succeeding downstream units, thus performing continuous and cascading reactions; (2) Enzyme-free Catalytic DNA circuits that possess a catalytic property, for which few input signals can be amplified to induce a large number of downstream reactions; (3) DNA circuits involving a biological enzyme: protein and DNAzyme, whereby DNA circuits can be implemented by multiple enzymetic mechanisms; (4) a DNA circuit on DNA origami, where the path-selectable molecular circuits and nanomachines can be realized through use of the shape diversity and spatial extensibility of DNA origami and (5) a DNA circuit combined with nanoparticles, wherein the dynamic self-assembly of nanoparticles can be precisely controlled by circuits to form specific nanostructures and perform biosensing.

## 2. The Research Studies on DNA Circuits

### 2.1. Cascading DNA Circuits In Vitro

The cascade effect is a signal transmission phenomenon, moving from the upstream to the downstream unit within an integrated system [49]. In a cascading DNA circuit, the DNA signal can be transmitted to a downstream reaction along a long-range signaling path. During the cascading reaction process, the free energy of the whole system gradually reduces toward a more stable energy state [50]. As a result, a cascading DNA circuit is a precisely designable information processing system that is able to produce the expected signal. Therefore, cascading DNA circuits are usually used to build continuous multiple-layer reaction systems [51], with the capacity to transmit molecular information and perform a computing function.

The Materials and Methods should be well thoroughly detailed so as to allow others to replicate and advance on the published results.

**Figure 1:**
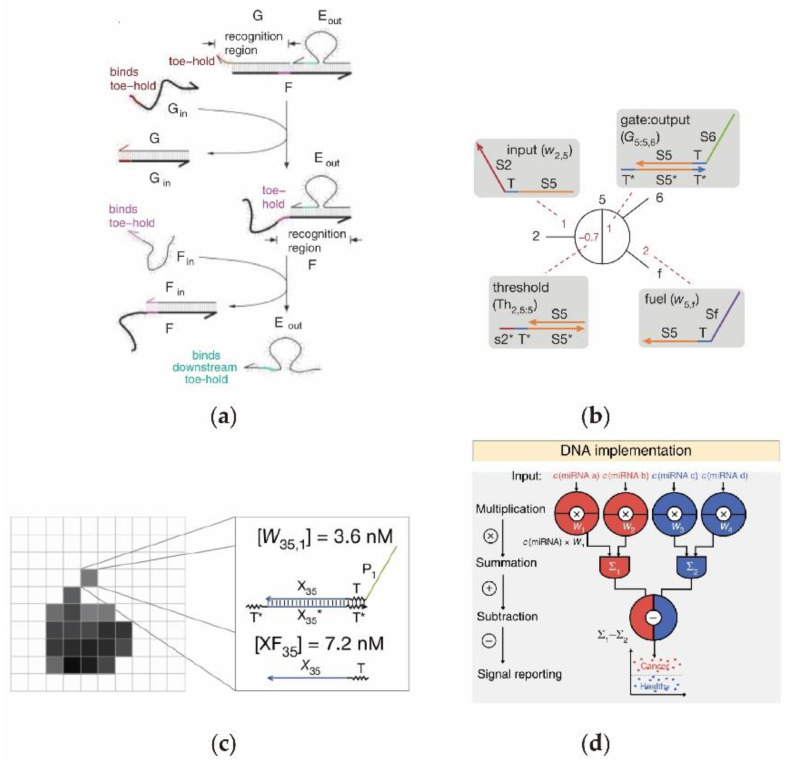
Cascade circuit to perform neural networks. (**a**) Schematic diagram of the DNA cascade circuit based on creating toehold sites. (**b**) Structure of DNA circuit neurons with weights and thresholds comprised of DNA strands, where multi-input and multi-output functions are performed. (**c**) Schematic diagram of the DNA neural network circuit to implement handwritten character recognition system. (**d**) The neural network computing model designed using the DNA cascade circuit to classify and identify Cancer.

Interestingly, one of the typical applications of a cascading DNA circuit is to construct a DNA computing system with continuous passing information. Imitating the modularization of electronic circuits, Seelig et al. [52] designed a cascading DNA circuit using ssDNA as the input/output signal through DNA strand displacement. The process is illustrated in Figure 1a. The output Eout is restricted in the double-stranded DNA structure because of its hybridization with substrate F at the beginning of the reaction. Once the input Gin is added to the system, it will immediately hybridize with G and expose the toehold site, thus inducing the hybridization between Fin and F to produce a displacement reaction that will release the output Eout. It is worth noting that the single stranded nucleic acid or microRNAs in this DNA-based digital logic circuit can be used as both the input and output, which places the digital logic circuit within the research scope of bioengineering. On the other hand, inspired by the cascading movement in living bacteria, Venkataraman et al. [53] constructed a DNA circuit-based molecular motor, in which a programmable and automatic movement can be implemented in vitro. However, the DNA computing unit, that has a simple design, is limited by its own computing power, so it is unable to realize more complex intelligent computing circuits. The neural network computing model has been widely applied in many instances that require complex computation in recent years, because of its special function of fitting arbitrary nonlinear curves and of storing a complex internal logic network. Interestingly, the neural logic possessing weight and threshold function can be regulated during the cascading reactions. Recently, Qian et al. [54] designed a molecule calculator with the function of computing the square root of binary data by designing a DNA cascade circuit based on the basic neural network model (Figure 1b). Afterwards, based on the principle of the construction of another Neuronal connection pattern, Cherry and Qian [41] designed a winner-take-all neural network realizing the digital pattern recognition function using a DNA circuit (Figure 1c). The digital pixels were represented by a DNA neural circuit, where the computing weights were determined using the DNA concentrations. Using the complex DNA neural network, the molecular pattern recognition function can be performed. Since the DNA neural network circuit has been demonstrated to perform complex logic operations at the gene manipulation level, the DNA circuit designed according to its model is also designed for a rapid and accurate diagnosis of cancer [55]. Figure 1d illustrates a DNA neural network circuit that uses miRNA in clinical serum samples to detect cancer. In this system, different kinds of miRNA from the clinical serum samples are first identified as input signals by the DNA neural network circuit, and are then sequentially implemented by DNA multiplication, summation and subtraction to achieve a rapid and accurate cancer diagnosis. With its flexible network structure and diversified computing functions, a computing platform with this type of DNA neural network has the opportunity to implement its unique advantages in medical diagnosis and large-scale data computing.

### 2.2. Enzyme-Free Catalytic DNA Circuit

In natural gene networks, protein enzymes are typically used as catalysts, while DNA seldom performs a catalyzing function. This means that the input DNA signals are continuously consumed in a normal noncatalytic DNA circuit reaction, thus leading to a limited reaction efficiency. In synthetic DNA circuit, by introducing a recyclable DNA as a catalyst the reaction efficiency is greatly improved, and only a small amount of input DNA is required to release a large amount of output DNA [56]. Therefore, a catalytic DNA circuit can be used to construct a more efficient artificial nucleic acid system without the use of a bioenzyme.

**Figure 2:**
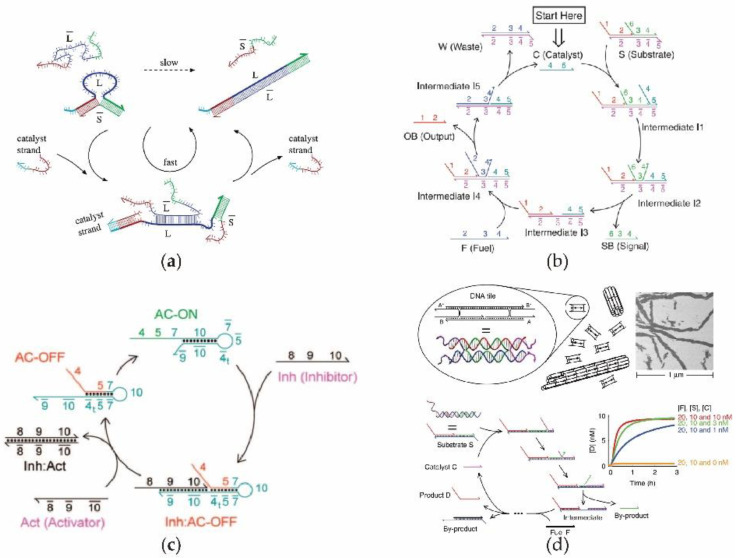
Schematic diagram of catalytic DNA circuit (**a**) The DNA catalytic circuit in which the catalyst can be used repeatedly in each reaction cycle. (**b**) Catalytic DNA circuit using single-stranded DNA as a catalyst, where DNA intermediates, output products, and signals are produced during each cycle with sufficient fuel DNA. (**c**) Catalytic DNA circuit using neck ring DNA as catalyst. During the catalytic process, the structures of neck ring DNA are dynamically changed. (**d**) Large-scale assembly of DNA title using DNA enzyme-free catalytic circuit.

In 2006, Seelig et al. [57] first proposed a DNA circuit with high catalytic efficiency based on DNA strand replacement by establishing a catalytic cycling unit. Figure 2a illustrates the operation of this enzyme-free catalytic circuit based on the principle of DNA displacement. Without a catalyst strand in the system, because the structure of $\overset{—}{S}$L itself is a relatively stable double-stranded structure, the input $\overset{—}{L}$ displaces the output $\overset{—}{S}$ from L at a slower rate. Once the catalyst strand is added to the original system, the hybridization between the catalyst strand and L will be triggered immediately to reduces the stability of SL, thus greatly increasing the reaction rate of $\overset{—}{L}$ to $\overset{—}{S}$ through DNA displacement. It should be noted that a single catalyst strand can be used repeatedly in each cycle of this enzyme-free catalytic reaction, as a small amount of the catalyst can result in the system possessing a high catalytic efficiency. Since the linear DNA structure is more stable and easily transformed, Zhang et al. [58] designed an enzyme-free DNA circuit with a high catalytic performance, using single-stranded DNA as a catalyst. As shown in Figure 2b, the DNA catalyst was used repeatedly in the catalytic cycle without being consumed, and a large amount of outputDNA was produced along with the generation of waste and intermediate products. It is worth noting that the reason that catalysis is continuous is not only because the catalyst is repeatedly used, but more importantly, an amount of fuel is consumed in each catalytic cycle. In other words, both the efficiency and rate of the catalytic reaction are largely dependent on the amount of available fuel. To dynamically control the catalytic circuit process, Zhang et al. [23] introduced an inhibitor DNA and activator DNA into the catalytic system. As shown in Figure 2c, in contrast to a simple linear ssDNA catalyst, a loop DNA catalyst can be used to perform the catalytic function, where the catalytic process can be dynamically switched according to the specific inhibitors and activators. By combining the enzyme-free catalytic circuit with DNA tile technology, David Yu Zhang et.al realized the rapid and large-scale assembly of micro-nanotubes [59]. The linear catalytic circuit in Figure 2d continuously generates the single-stranded DNA required to assemble large-scale nanotubes, thereby achieving the function of efficiently increasing the size of nanotubes within a short period. Because of its efficient and stable catalytic ability and simple circuit structure, this type of enzyme-free catalytic circuit is widely used in DNA circuits that are required to amplify specific signals. More importantly, since the three kinds of important state quantities (catalyst, output, and fuel) in these catalytic systems can be supplied by other independent DNA circuits or vice versa, the catalytic system can cascade with other independent DNA circuits to amplify the specific signals. This is of great significance for the accuracy and sensitivity of signal detection.

### 2.3. DNAzyme-Based DNA Circuit

DNAzyme is a specific single-stranded DNA fragment with a catalytic cutting function, high catalytic activity, and specific recognition capacity. One of its most important roles is to cleave the RNA site via esterification. By taking advantage of this property, the DNA circuit can directly employ DNAzyme to accurately control its circuit structure and signal transmission mode [60,61,62]. On the other hand, the structure and activity of DNAzyme can be precisely regulated, thus providing the stronger controllability for DNA circuits [27,63,64,65,66,67,68].

**Figure 3:**
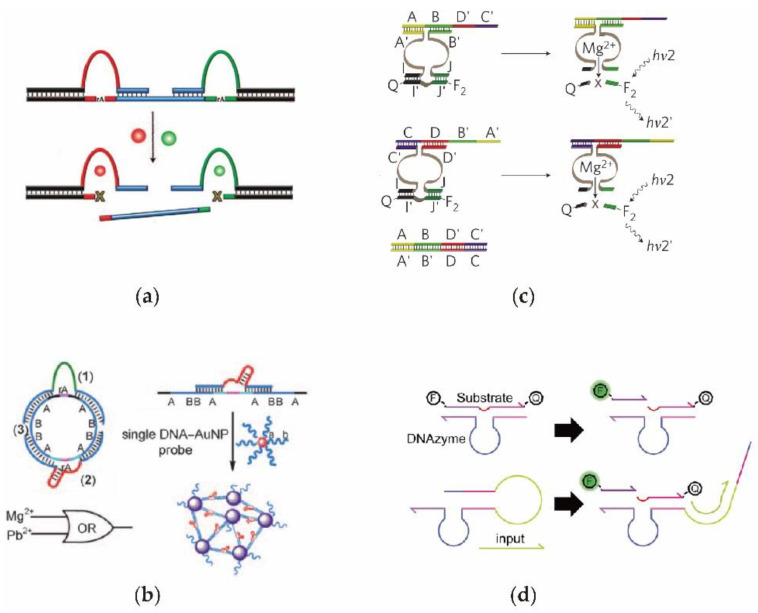
Schematic diagrams of DNAzyme-based synthetic DNA circuit. (**a**) DNAzyme DNA circuit able to recognize metal ions. (**b**) DNAzyme DNA circuit to realize controllable self-assembly of nanoparticles to construct logic gates. (**c**) Editable DNA circuit constructed by the combinations of variable DNA scaffolds. (**d**) Controllable DNAzyme switches with repeated open/off control.

Utilizing a metal-ion ion dependent DNAzyme, Moshe et al. [69] constructed a DNA circuit that was regulated by uranium dioxide and magnesium. As shown in Figure 3a, only once both DNAzymes were activated simultaneously could the target DNA sequence be released. In addition, a simple Boolean logic operation was implemented by detecting the presence of uranium dioxide and magnesium in the reaction environment. The success of the DNAzyme logic circuit demonstrates that DNAzyme circuits have the potential to be scaled up to imply complex computational functions. Moreover, by combining gold nanoparticles with DNAzyme, Bi et al. [70] designed a DNAzyme logic circuit characterized by colorimetic and UV/vis detection. As illustrated in Figure 3b, circle DNA with different RNA modification sites can be cut into several DNA fragments by the DNAzyme, resulting in the aggregation of gold nanoparticles.

The potential of the upstream output signal being successfully transmitted to the downstream as an input signal without a signal leakage in the process of transmission remains a major challenge in DNAzyme cascade circuits. Unlike the traditional DNAzyme with fixed structures, Elbaz et al. [71] constructed a DNA circuit with a controllable DNAzyme by splicing the structure into a hybrid structure (Figure 3c). Therefore, the inputs and outputs in the overall system consist of only simple single-stranded DNA, contributing to a strong editable and extensible circuit performance, and achieving modular DNAzyme-based cascading signal conduction. To extend the control dimension of this circuit, Elbaz [72] established a DNAzyme system based on pH regulation. The signal catalytic function of DNAzyme provides the advantage reusability in the process of RNA cleavage, however, it also causes the DNAzyme-based circuit to become uncontrollable, which greatly limits the time domain characteristics of the circuits. Compared with the previous unchangeable DNAzyme, Harding et al. [73] designed a DNA circuit using the DNAzyme with a dynamic switching ability (Figure 3d). Therefore, the activity of the DNAzyme can be well regulated using an additional input DNA signal.

### 2.4. Protein Enzyme-Assiste d DNA Circuit

A biological enzyme is a type of biomolecule that can specifically recognize the substrate to perform its catalytic function [74]. According to their catalytic reaction properties, biological enzymes can be generally divided into categories such as oxidoreductase, transferases, and hydrolases. Due to its high specificity, strong catalytic ability, and fast reaction speed, the enzyme-assisted DNA circuit is suitable for building a more intelligent biological computing system. Recently, biological enzymes have been used to build functional DNA circuits [75,76,77].

**Figure 4:**
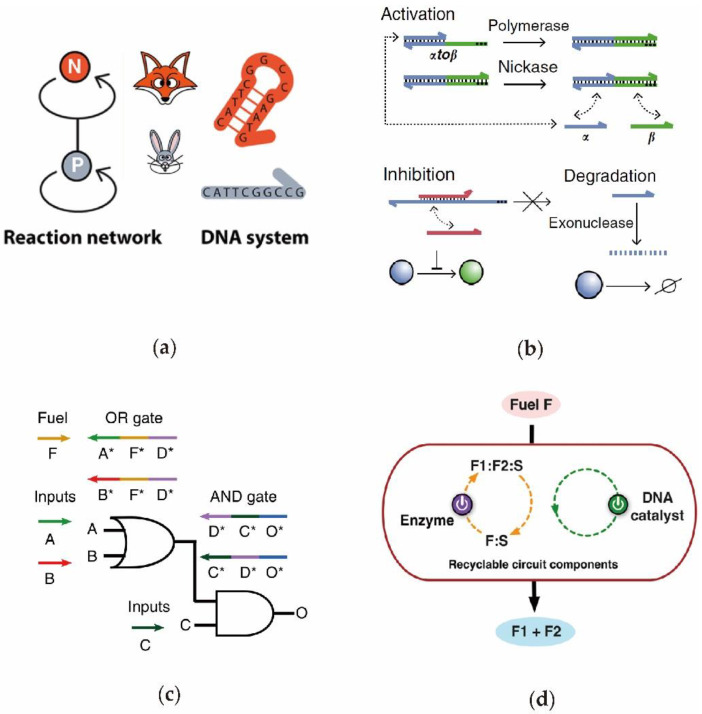
Designs of biological enzyme-involved DNA circuits. (**a**) Schematic diagram of the DNA logic gates using strand displacement polymerase. (**b**) Highly efficient dual-catalytic recyclable DNA circuit driven by nicking enzyme and DNA catalyst. (**c**) Schematics for the oscillating DNA circuit based on protein enzyme. (**d**) The enzyme-toolbox-directed DNA circuit with activation, inhibition, and subtraction functions.

Through their simulation of ecosystems, Fuji et al. [5] designed an enzyme-assisted DNA circuit with oscillating and competitive functions (Figure 4a). In the system, both the predator P and prey N were dynamically generated and degraded in the presence of the polymerase, nicking enzyme, and exonuclease. Therefore, through the rational design of the concentrations of reactants, DNA circuits with different oscillation and attenuation periods can be established to realize the expected circuit functions. In 2017, inspired by signaling networks in living cells, Lenny et al. [78] designed an enzyme-driven DNA circuit that can dynamically regulate the signal strength during a reaction (Figure 4b). The researchers applied this toolbox to various DNA circuits to realize the bionic electronic functions by using DNA as the information carrier. In the study, the DNA circuit can perform the functions as converter, memory, and inverter. The results indicated that the bioenzyme-assisted DNA circuit has a gene network regulation capability. In order to resolve the issues of a slow reaction speed and the high complexity of the system in the DNA circuits, Song et al. [42] established a logic DNA circuit composed of simple ssDNA and polymerase-triggered DNA strand displacement (Figure 4c). Significantly, since the DNA circuit consists of only single-stranded DNA, the problems of signal leakage and signal reset are resolved well. In addition, the reaction time was considerably reduced as a result of the rapidity of the enzyme-driven strand displacement. Through the combination of the enzyme- and entropy-driven DNA catalytic reaction, Zhang et al. [79] constructed a dual-catalytic recyclable DNA circuit (Figure 4d). Compared with the previous catalytic DNA circuit, whereby the product could not continue its participation in the consecutive catalytic cycle, the reaction product in the dual-catalytic DNA circuit did not become a useless waste product, instead, it was reactivated to participate into the reaction to enhance the fluorescence signal. In conclusion, compared with the toehold-based DNA circuit, the protein enzyme-assisted DNA circuit has clear advantages in its reaction rate, circuit design difficulty and signal modulation diversity. More importantly, the required base sequences of input and output DNA can be designed to be completely orthogonal. Thus, as opposed to a protein enzyme-assisted DNA circuit, the signal leakage problem, in a toehold-based DNA circuit, is mitigated.

### 2.5. The DNA Circuits on Origami Surface

A DNA origami assembly was developed by Rohemund in 2006, in which hundreds of short DNA fold with a M13mp18 scaffold to construct various nanometer shapes, such as squares, circles, and triangles. As the surface of DNA origami can modify DNA in a practicable way, DNA circuits can be constructed onto the origami surface to achieve vector-based logic operations and robot movements [43,80,81,82,83,84]. Such an on-surface DNA circuit operation is a new method of gene regulation, exhibiting the properties of specific spatial designs and modular constructions.

**Figure 5:**
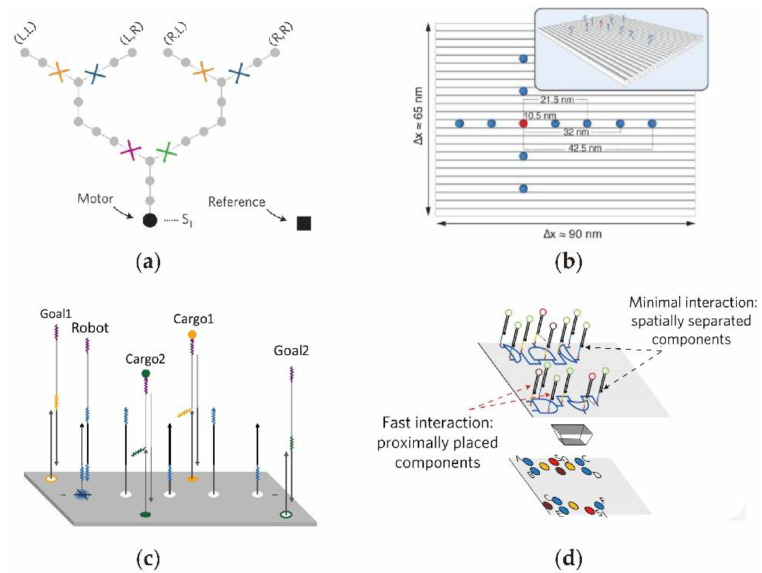
Design of DNA circuits on origami surface. (**a**) DNA walking motor that can follow the path on the DNA origami surface. (**b**) On-surface DNA circuit that can establish signal transmissions with addressable positions. (**c**) DNA transport cargos on origami surface. (**d**) On-surface DNA logic gates.

As shown in Figure 5a, by utilizing synthetic molecular motors that move autonomously along liner tracks, Shelley et al. [85] designed a programmed network system on the surface of a rectangular DNA origami. After moving, the majority of motors can follow the correct path to the predesigned target aim. This programmable on-surface DNA circuit motor has the potential to build complex and controllable information transmission systems. In 2014, to investigate the kinetic and efficiency of on-surface DNA circuit, Teichmann et al. [86] installed DNA circuit units of signal sender and receiver on an origami surface (Figure 5b). In the on-surface circuit, the sender unit was situated in the middle (red color point) of the origami platform and the receiver units were distributed evenly on the plane around the sender. Interestingly, researchers can regulate the signal transmission by adjusting the distributions of gate units, so as to optimize the efficiency and accuracy of the on-surface DNA circuits. In addition, the precise arrangement of on-surface circuit is of great significance for DNA-based memories and information processing. In 2017, Winfree developed a DNA robot with the ability to carry cargo in sequence on the origami surface by designing a randomly moving DNA circuit [87] (Figure 5c). After the random movement, the cargo molecules distributed on the surface can be arranged by establishing a DNA circuit on the DNA origami surface. Because the cargo molecule information can be predesigned, this DNA circuit robot was able to perform molecular information transmission on the surface. Similar to the transmission of electrical signals in a silicon-based circuit, Gourab et al. [49] constructed a surface logic DNA circuit whereby the signal transmission path can be programmed to realize the directional logic operation as shown in Figure 5d. In the study, different signal transmission routes were performed by a variety of DNA circuits and multiple levels of information interactions occurred among the logic gates on the surface. Notably, with its capability to implement almost arbitrary logic gate functions, the on-surface DNA circuit can be used to solve more complex logic operations.

### 2.6. The DNA Circuits Combined with Nanoparticles

Compared to other macroscopic materials, nanoparticles possess the advantages of well-controlled sizes and shapes [45,88,89], visible spectroscopy characteristics [90,91,92,93], and multiple chemical modifications. Therefore, nanoparticles have been widely used in multiple disciplines, e.g., in medicine, bioengineering, and molecular signal detection [94,95,96,97,98]. Recently, combined with a DNA circuit, nanoparticles have been widely applied to perform the functions of biosensing, molecular signal detection, and DNA computing [99,100,101,102].

**Figure 6:**
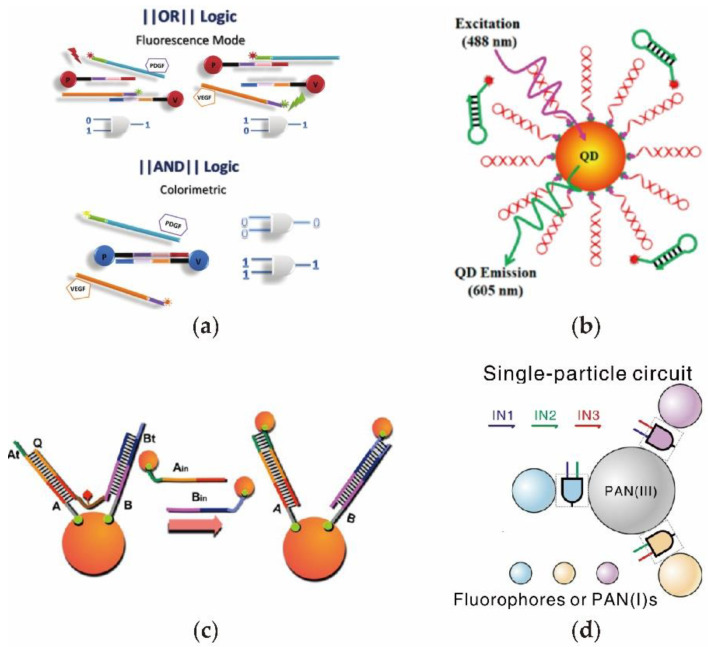
Design of DNA circuits combined with nanoparticles (**a**) DNA circuit directing the aggregation of gold nanoparticles to detect the target protein. (**b**) Catalytic DNA circuit on quantum dots to detect low-concentration target microRNA. (**c**) Multiple-layer DNA circuit on gold nanoparticles. (**d**) Programmable nanoparticle-assisted DNA logic circuit.

After Mirkin et al. [103] proposed a method of binding nucleic acid onto gold nanoparticles in 1996, scientists have diverted their attention to the area of DNA circuits combined with nanoparticles. In 2007, Zhao et al. [104] designed a DNA circuit to detect metal ions and enzyme activities based on colorimetric change induced by nanoparticle aggregations. To obtain reliable and rapid sensors for diagnostic applications, Tan et al. [105] constructed a DNA circuit for detecting target protein-combined gold nanoparticles with dye-labeled aptamer probes (Figure 6a). Once the nanoparticles aggregated as a result of the recognition between DNA probes and targets, the color of the solution rapidly transitioned from red to blue, thus indicating the positive sensing result. In addition, sensing low-concentration targets with the use of biomarker detection remains a challenge for clinical diagnosis. In 2019, Ma et al. [106] built a self-assembly nanosensor composed of semiconductor quantum dots (QDs) to detect microRNA without using biological enzymes (Figure 6b). In a DNA circuit reaction, microRNA can serve as the catalyst to trigger the catalytic cycle, thus producing the dual-labeled DNA duplexes on the QD to cause FRET. The detection of microRNA was achieved by using a nanoparticle-based DNA circuit. By combining gold nanoparticles with a DNA logic gate, Yang et al. [107] designed a DNA circuit using fluorescent beacon probes and transmission electron microscopy to obtain the output signals (Figure 6c). In the reactions, the circuit was operated on through a surface of gold nanoparticles to obtain the basic logic gates. By using biopolymers to program biomolecular interactions, Yao et al. [108] constructed a DNA circuit to achieve the programmable self-assembly of gold nanoparticle with a different chirality property (Figure 6d). Furthermore, the Boolean logic function was also implemented by arranging programmable nanoparticles.

## 3. Conclusions

A nanotechnology-based synthetic DNA circuit has been well developed in recent years involving many research fields, such as molecular biology, mathematics, and information science. Furthermore, the DNA circuit has been widely applied in gene regulation, biosensing, diagnosis, and DNA computing. The flexible and diverse construction methods of DNA circuits in particular, provide additional and efficient routes for gene network regulation and gene detection. Meanwhile, the programmable and cascaded properties of the DNA circuits also facilitate the construction of a DNA computing system designed to solve complex problems, e.g., the DNA neural networks with genotyping and pattern recognition function are realized via large-scale multilevel cascade DNA circuits [41].

Notably, a signal amplification function is an important step in DNA circuits to implement molecular signal amplification. Firstly, based on the enzyme-free, entropy-driven mechanism, a synthetic DNA circuit can perform a catalytic function, thus enabling the detection of weak signals. On the other hand, combined with a biological enzyme, complex DNA circuits have also been developed to achieve multilevel molecular signal amplification, transmissions and logic operations. It should be noted that different types of enzymes can be simultaneously used, even within one system. For example, complex dynamic bistable signaling systems are constructed by using ligation enzymes and cleavage enzymes simultaneously [5,78].

Moreover, via the highly programmable and designable properties, a synthetic DNA circuit also can be implemented through various modes of nanomaterial assembly and abiotic signaling, thereby realizing the diverse functions of molecular sensing and biological computing. Considering the ease with which specific chemical groups can be modified on DNA molecules, a variety of nanomaterials, such as gold nanoparticles and DNA origami, can be assembled in a programmable way to realize the required DNA circuit operations [81,83,104,106]. On the other hand, the functions of the DNA circuit are enhanced through their combination with some abiotic signals. For instance, some DNA circuit-based nanosensors and logic gates can be regulated by the signals of metal ions, pH, and light [7,72,108].

Although DNA circuits have engendered remarkable progresses in recent years, there are still some critical aspects that need to be improved upon to broaden the related application fields. The most important aspect is the signal leakage and distortion that exists in various complex DNA circuits [41,54]. The primary reason for this phenomenon is that some redundant base complementary pairings easily emerged during the circuit operations. At present, the design methods implemented to program DNA sequences are mainly based on the combinations of a manual design and simple software. Therefore, it is still difficult to completely eliminate these interference signals using pre-established methods, and a more automatic and universal sequence design platform is eagerly desired in the future. In addition, another important consideration to account for is the method by which to ensure the DNA circuit is reusable, thereby promoting its practical applications. Compared with the repeated electronic circuits, DNA circuits cannot be reused after completing the first complete operation [42,55,108], which inhibits the subsequent development of more advanced intelligent DNA circuits. Specifically, in large computing DNA circuits, it is important to establish a reusable circuit mechanism, to meet requirements of frequently adjusted circuit parameters and to test the structural stability of the system. In fact, many studies have been undertaken to improve the repeatability of the DNA circuits. More recently, Zhang et al. reported a nicking-assisted recycling strategy for implement reusable DNA circuits to achieve a repeatability of over 20 times during one reaction cycle [79].

In general, a synthetic DNA circuit, as a recently developed molecular intelligent nanosystem, serves as an artificial bridge to communicate between the artificial molecular systems and natural gene networks. Through the use of precise control mechanisms and cascading modulation, the DNA circuit can programmatically regulate the functions of the molecular systems and interact with natural gene networks to achieve accurate gene regulation. It is believed that in the near future, with the assistance of more advanced nanotechnologies and through interdisciplinary research fields, the synthetic DNA circuit will provide novel applications in the areas of gene regulation, diagnosis, biosensing, and bioinspired computing.
